# Capillary Uptake Monitoring in Lime-Hemp-Perlite Composite Using the Time Domain Reflectometry Sensing Technique for Moisture Detection in Building Composites

**DOI:** 10.3390/ma13071677

**Published:** 2020-04-03

**Authors:** Przemysław Brzyski, Zbigniew Suchorab

**Affiliations:** 1 Faculty of Civil Engineering and Architecture, Lublin University of Technology, Nadbystrzycka Str. 40, 20-618 Lublin, Poland; 2 Faculty of Environmental Engineering, Lublin University of Technology, Nadbystrzycka Str. 40B, 20-618 Lublin, Poland; z.suchorab@pollub.pl

**Keywords:** hemp-lime composites, capillary uptake, time domain reflectometry, material moisture, thermal conductivity

## Abstract

The use of waste plants in the production of building materials is consistent with the principles of sustainable development. One of the ideas involves using hemp shives as an aggregate for the production of a composite used as a filling of the timber frame construction of the walls. The most important disadvantage of using the building materials based on organic components is their susceptibility to the water influence. The wall material is exposed to rising groundwater. The research part of the paper presented the preparation method and the investigation of the hemp-perlite-lime composites. Flexural and compressive strength, apparent density, total porosity, thermal conductivity, and mass absorptivity were examined. The main research part pertained to the analysis of capillary uptake occurrence in the composites, being the important phenomenon present in the external walls. The study on this phenomenon was carried out using the technique of indirect moisture evaluation—Time Domain Reflectometry (TDR). The indirect readouts were additionally verified with the traditional evaluation using the gravimetric method based on the PN-EN 1925 standard. The study proved that the tested composites were characterized by low apparent density, thermal conductivity, strength parameters, high total porosity, and mass absorptivity. The partial replacement of hemp shives by expanded perlite had a beneficial effect on the tested properties of composites.

## 1. Introduction

Within recent years, numerous scientific reports on the utilization of vegetal materials in the building sector have been published. It is a consequence of the growing interest of potential investors in natural building. Such kind of houses, besides their ecological aspects (that are not influencing regular lives), is also beneficial for the residents due to the healthy microclimate. The high porosity of vegetal materials also influences the appropriate thermal insulation parameters [1]. The hygroscopic character creates the ability to adjust the moisture levels in the indoor rooms. The walls that are made of straw or hemp composites are properly combined with clay, which is usually applied for wall plastering. Clay is also a favorable material for people with allergic diseases because it has the ability to absorb various types of dust. Additionally, the above-mentioned natural materials are characterized by high diffusivity permeability, which protects the walls from the vapor condensation inside the envelope or on internal surfaces, which would lead to mold development. Scientific research is currently being conducted on the different natural materials that could be utilized as building materials. The examples include cut sunflower stalk mixed with gypsum binder [2] as insulation board, sisal fibers [3] as scattered reinforcement, flax [4] as an element of chipboards, hemp fibers and shives [5] in mortars, flax or hemp fibers as thermal insulation [6], but also ligneous parts of stalks cut into shives of hemp used as aggregate for the composites based on lime binder [7,8].

The most important disadvantage related to the usage of building materials based on organic components is their susceptibility to the water influence [9]. Natural protection over extensive moisture in the boundaries that are built of natural, vegetal materials usually constitutes the thick layers of clay or lime plaster. In dry ambient air, these materials can absorb the water from the wall structure and transport it to the air [10].

In the case of the building materials applied for walls construction, an important feature is the ability of capillary uptake of groundwater. The capillary forces depend on the quantity, dimension, and orientation of the open pores in the material [11]. The visible consequence of the capillary uptake phenomenon is the moisture present at the bottom levels of the walls, reaching various heights depending on the material parameters [12]. Besides the visual aspects of capillary uptake, the decrease of thermal parameters of material [13] and finally rotting of the wooden elements of the wall should be mentioned as well. In order to prevent this phenomenon in the newly built boundaries, horizontal waterproofing layers should be applied between the foundation and the wall.

The article presented the results of the research on the capillary uptake ability of water by the composites comprising the wastes from local hemp. The moisture performance of the three types of composites that differ in the ratio of binder to filler and have different amounts of two kinds of fillers (hemp shives and expanded perlite) was presented. The capillary uptake process was examined with two methods: gravimetric (based on the PN-EN 1925 standard [14]) and the reflectometric technique of indirect moisture detection—Time Domain Reflectometry (TDR). Additionally, for wider material recognition, the basic properties of the composites, such as the flexural and compressive strength, apparent density, total porosity, thermal conductivity, and mass absorptivity, were examined as well.

Time Domain Reflectometry (TDR) is a technique applied to determine the apparent permittivity of porous materials with the measurement of the electromagnetic pulse propagation time along the rods of the measuring probes. The essentials of the technique are described in detail in the following articles [15,16]. Apparent permittivity is a measured parameter, describing the behavior of the molecules under the alternate electromagnetic field and the dissipation of the energy after the field is released. This measure can be determined using the TDR equipment, and its value depends on the moisture of the material. According to the physical and empirical models available in the literature [15,17,18,19,20,21,22,23], its value depends on the shares of particular phases forming the material. Water has the most dominant influence on the TDR readouts due to its polar particle character and the fact that its apparent permittivity value amounts to about 80 [-]. It is significantly greater than the apparent permittivity of the solid 3–10 [-] and air 1 [-] phase [24,25,26,27]; the difference contributes to the potential of the TDR as a measuring technique for moisture estimation of many porous media.

The TDR technique has been intentionally applied in the field of soil science to evaluate the moisture content of the soils. However, several approaches to utilize its potential for evaluating the moisture of construction materials have been noted in the scientific literature. Most of the investigations on building materials are conducted using the traditional, invasive probes used for soils [28,29,30,31,32]. Several attempts have been also made to evaluate the moisture of building materials by the co-authors of this article [33,34]

## 2. Materials and Methods

### 2.1. Description of Materials

The research was conducted on the composite based on a modified lime (L) binder and aggregate substitute involving hemp (H) shives and expanded perlite (P) (Three recipes that differed in the proportion of the applied compounds were investigated. The first recipe (HL1) contained only hemp shives as an aggregate substitute, the other two compositions (HLP1 and HLP2) besides shives also contained expanded perlite. The same binder was used in all recipes. It was a mixture of hydrated lime CL-90s (Lhoist, Tarnów, Poland) (70% of binder dry mass) with metakaolin (Astra Polska, Gdańsk, Poland) and Portland cement CEM I 42.5R (Cemex, Chełm, Poland)—both 15% of binder dry mass. This mixture created a binder, which was called artificial hydraulic lime. Hydrated lime was an air binder with low strength parameters. However, it was characterized by high vapor permeability and resistance to biological corrosion due to high pH value. Metakaolin was a highly reactive pozzolanic material, which was formed in the kaoline clay calcination process under controlled temperature conditions of around 600–850 °C. Its main ingredient was hydrated aluminum silicate Al_2_Si_2_O_5_(OH)_4_. This additive has been used in other studies of the hemp-lime composite [35].

The formulas of the tested composites are presented in Table 1 (amounts by weight per 1 m^3^) and in Table 2 (proportions by weight).

**Figure 1:**
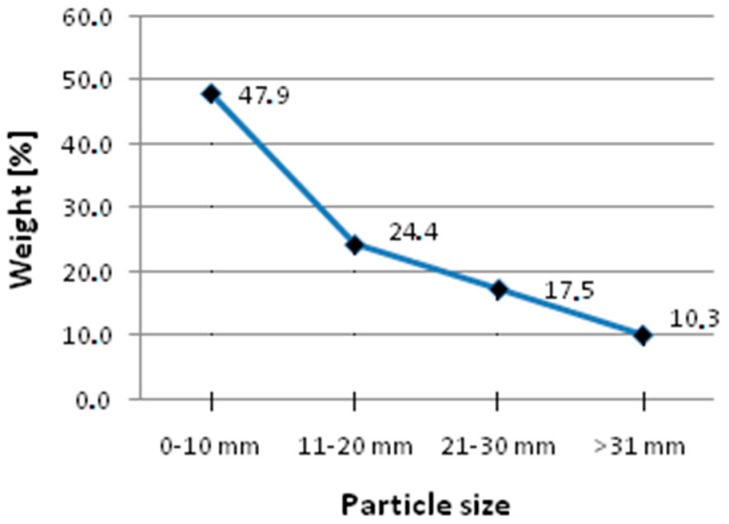
Grading curve of the hemp shives used in the study.

The hemp shives applied in all four recipes were Polish type of the industrial hemp named Białobrzeskie (the Institute of Natural Fibers and Medicinal Plants, Poznań, Poland). It was adapted to the local, Polish soil-climatic conditions. The hemp filler was characterized with high total porosity, about 90%, which has also been confirmed by literature (90.1 ± 0.5% [36]), and low apparent density, about 130 kg/m^3^, as in other studies (about 125 ± 9 kg/m^3^ [36]). The applied mixture of hemp shives was characterized by a large diversity in length, width, and thickness of fractions. The weight shares of particular fractions are presented in Figure 1. A mixture of shives also contained small amounts of hemp fibers and dust.

**Figure 2:**
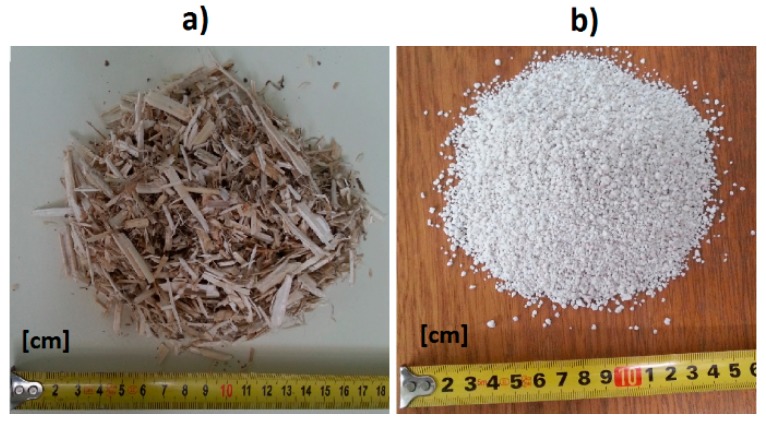
Fillers in composites: (**a**) hemp shives, (**b**) expanded perlite.

EP180 (Perlit Polska, Puńców, Poland), second class perlite with granulation of 0–4 mm, was applied for the HLP1 and HLP2 composites. The perlite content in relation to the binder content was from 20% to 30%. The compression strength of the perlite was 0.14–0.40 MPa, bulk density 80–100 kg/m^3^, and heat conductivity coefficient 0.042 W/(m·K) [37]. The perlite additives were applied in order to achieve a compacted structure by filling the air gaps between the randomly arranged hemp shives. Additionally, it was expected that perlite additives should decrease the intensity of the capillary uptake phenomenon and minimize the threat of mold and fungus development. The fillers used in the tests are shown in the figure below (Figure 2).

Water was also applied as a mixture compound with the precisely established amounts for the hemp-lime composites. Only the minimal possible amount of water necessary for binding was used because the excessive water would lead to an increase in drying time. Under the extreme circumstances, it might cause the disintegration of mixture compounds and thus storage of the binder in the bottom parts of wall boarding or escape of the binder through the leaks in the boards.

### 2.2. Sample Production Process

The sample preparation process involved the binder mixing with water and gradual supplementation of the liquid binder to the initially mixed fillers, maintaining the constant mixing. The mixing procedure lasted until the hemp shives and the perlite were covered with a binder, and the mixture obtained a homogenous consistency. A similar procedure of mixing could be found in the following papers [38,39]. On the other hand, Cerezo and Nguyen, in their research, firstly soaked the hemp shives and then supplemented them to the mixed binder materials [40,41]. Next, the samples were formed. Due to the presence and the shape of the lightweight filler, mixture compaction under gravity was not possible. The samples were thickened by hand beating with a wooden peg with a diameter of 30 mm.

### 2.3. Determination of Composite Parameters

#### 2.3.1. Apparent Density and Porosity

In order to parameterize the material, its basic physical features were determined. Apparent density, specific density, and open and total porosity of the composites were measured. The measurements were conducted according to the PN-EN 12390-7 standard [42]. Total porosity was estimated as a ratio of the total volume of open and closed pores per sample volume. 

#### 2.3.2. Flexural and Compressive Strength

The strength tests were conducted using an MTS 810 apparatus (MTS System Corporation, Eden Prairie, MN, USA) with the range of load between 0–100 kN. The flexural strength was estimated using samples with a dimension of 100 × 100 × 500 mm. The compressive strength was determined on samples with the following dimensions 150 × 150 × 150 mm. Displacement of the compressing head was set to 1 mm/min (flexural strength test) and 3 mm/min (compressive strength test). The average of three attempts was assumed as the measurement readout.

#### 2.3.3. Thermal Conductivity

The thermal conductivity determination was conducted using the samples with the dimensions of 300 × 300 × 50 mm after 28 days of maturing. The measurement was conducted using the FOX314 plate apparatus (TA Instruments, New Castle, DE, USA). Before the experiment, the samples were dried to constant mass. In order to achieve the average temperature of 12.5 °C, the heating plate temperature was set to 25 °C and the cooling plate to 0 °C. The result of the research was an average of the heat conductivity coefficient of the composite achieved in three attempts for each recipe.

#### 2.3.4. Mass Absorptivity

The absorptivity is the ability to absorb water at atmospheric pressure. The mass absorptivity tests were conducted according to the PN-EN 13755:2008 [43] standard on three 100 × 100 × 100 mm specimens for each recipe. The samples were completely submerged in water. Its result was the ratio between the mass of the absorbed water and the mass of the dry sample. 

#### 2.3.5. Capillary Uptake (Standard Method)

This experimental test was conducted in accordance with the PN-EN 1925 standard [14]. Three samples of each recipe, with the dimensions of 150 × 150 × 150 mm, were placed in contact with water at a depth of approximately 10 mm and maintained at a constant level by using a tank water system. In the determined intervals of time, the increase in the mass of samples was noticed. In this way, the amount of water absorbed was determined. An increase of the mass of the samples was measured in the following intervals of time: 1 h, 3 h, 6 h, 12 h, 24 h, 3 d, 4 d, 6 d, 10 d.

#### 2.3.6. Capillary Uptake (TDR Method)

The capillary uptake experiment was conducted using Time Domain Reflectometry (TDR) method by applying the following equipment:

- Set of TDR field probes for moisture determination (FP/mts, EasyTest, Lublin, Poland),
- TDR multimeter with multiplexer (LOM, EasyTest, Lublin, Poland),
- PC for multimeter control and data processing,
- Software to control TDR multimeter and post-process the obtained data,
- Dryer (Memmert VO-500),
- Water container,
- Samples of the examined composite with the dimensions of 150 × 150 × 150 mm.

**Figure 3:**
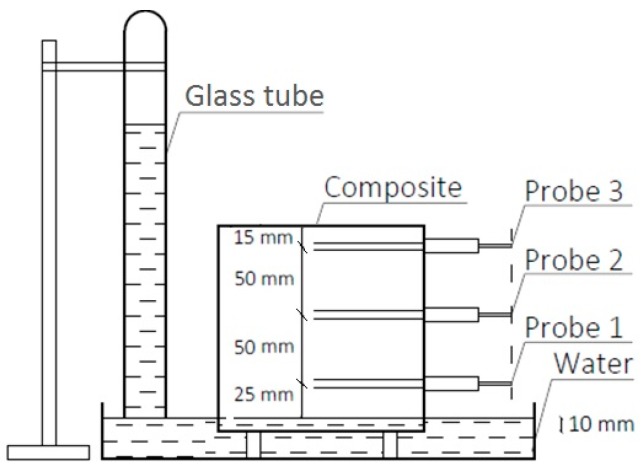
Schematic view of the measuring setup.

**Figure 4:**
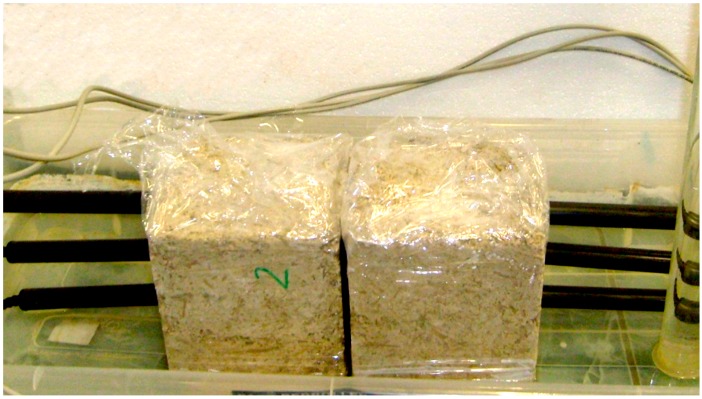
Photography of two samples examined with the experiment with installed Time Domain Reflectometry (TDR) FP/mts probes.

The measurement was conducted on 3 samples for each recipe (nine samples were tested). Three TDR probes were inserted in vertical line into each sample according to the figures below (Figure 3 and Figure 4) at the following heights 25, 75, and 125 mm above the water level.

The samples were placed in a water container with a constant water table. The bottom surface of each sample was placed 10 mm below the water level. In order to prevent the water absorbed by samples from surface evaporation, the samples were covered with anti-diffusive foil. The TDR probes were inserted horizontally according to the compaction direction, which was simulating a building barrier made of composite placed monolithically in the wall formworks. The duration of the experiment was set to 44 days. The time step was set for 15 min. The experiment was conducted under laboratory conditions at a temperature of 22 ± 2 °C and relative humidity of 60 ± 5%.

During the experiment, the values of the apparent permittivity were read by means of the TDR equipment. They were later recalculated into the volumetric water content using semi-empirical Malicki’s calibration model [23]:

(1) $$\theta =\frac{\left(\varepsilon^{0,5}-0.819-0.168\rho -0.159\rho^{2}\right)}{7.17+1.18\rho }$$

where *θ*—volumetric water content (cm^3^/cm^3^); *ρ*—bulk density of the composite (g/cm^3^); *ε*—apparent permittivity value measured using TDR (-).

According to the literature sources, the applied calibration model is efficient in the moisture evaluation of the materials with the various features of the solid phase [27], and the measurement uncertainty is about 0.02 cm^3^/cm^3^ (2 vol %) [44,45,46].

## 3. Results and Discussion

Table 3 presents the results of individual tests (averaged values) pertaining to the basic mechanical and physical properties of the composites used in the experiment. The analysis of the results is presented in the following subsections.

### 3.1. Apparent Density and Porosity

The apparent density of the examined composites was between 417.5 and 503.1 kg/m^3^ (Table 3). According to the conducted experiment and literature review, it mainly depends on the method of compaction [47,48] and the proportion between the binder and the aggregate. Together with the increase of the binder share in the mixture, the density of composite arose, which could be also confirmed by the following literature reports [48,49].

There is also a strict dependence between volumetric density and apparent porosity, which comes directly from its definition. The high porosity of hemp shives, amounting to about 60%, and their position during compaction influenced high total porosity of a composite—74.4–79.3% as well as open porosity between 49.9% and 57.8% (Table 3). According to Rahim’s research [50], the porosity of hemp composites is comparable—the composite with a volumetric density of 478 kg/m^3^ indicates total porosity to be equal to 76.4% and open porosity 49.9%. The total porosity of hemp shives with a volumetric density of 125 kg/m^3^ equals to 90.1% with the shives fraction of about 5 × 5 × 15 mm. In a hardened composite, it is possible to distinguish pores, being the consequence of the uneven position of shives in the material and those present between hydrates in the binder matrix [47], but also the pores present in the shives structure.

According to [1], for the composites applied as a roof thermal insulation with the apparent density of 258 kg/m^3^, it is possible to obtain porosity of 84.9%, and by the increase of density to 457 kg/m^3^, required to use composites for prefabrication, porosity decreases to 72%. In another research, Collet [1] presented that the composite fabricated for walls rising using spraying revealed the total porosity of 79% and open porosity below 70% with the densities of 430–460 kg/m^3^. The research presented in [51] has investigated the composites with densities of 256–460 kg/m^3^ and porosities of 80–72%, respectively. 

### 3.2. Flexural and Compressive Strength

The average value of the flexural strength of composites reached from 0.16 to 0.23 MPa and compressive strength between 0.55 to 0.78 MPa (Table 3). A hemp-lime composite can be applied to fill the timber frame construction. It is a self-supporting material, which means that it supports its own weight and also stiffens the timber construction [52]. The increase of strength is directly combined with the decrease of other important physical parameters, such as density rise, thermal resistance decrease [49]. According to the conducted research, as well as the literature reports, the parameter of the composite, which influences strength to the greatest extent, is the binder share in relation to the aggregate [48,49]. The highest compressive strength was reached by the composites with a weight ratio between binder and aggregate 2:1. Those were HLP1 (0.78 MPa, 503.1 kg/m^3^) and HL1 (0.66 MPa, 497.9 kg/m^3^).

### 3.3. Thermal Conductivity

**Figure 5:**
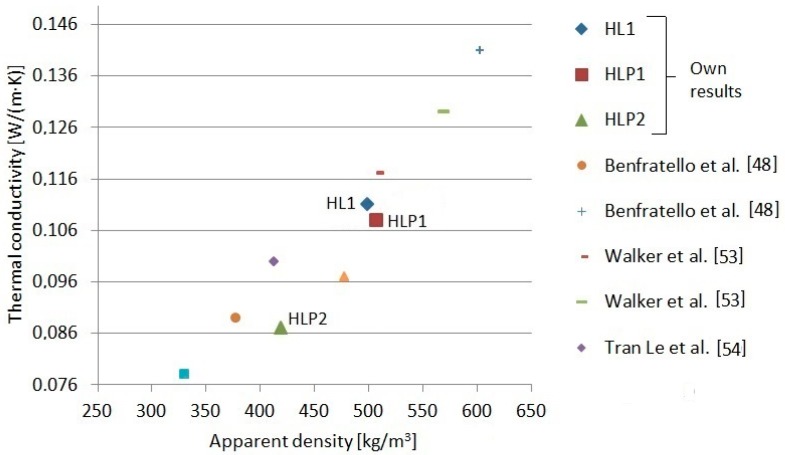
Relationship between the lambda coefficient and the apparent density of the hemp-lime composites [48,53,54].

The thermal conductivity coefficient of the examined composites comprised of values between 0.087 W/(m·K) and 0.111 W/(m·K). The value of this parameter depended mainly on the ratio between the binder and the filler, which was 2:1 and 1.5:1. The highest value of the λ coefficient was measured for the HLP1 composite with the highest apparent density equal to 503.1 kg/m^3^. On the other hand, for the HLP2 composite with the highest porosity equal to 79.3%, the heat conductivity was the lowest. What is important that the partial replacement of the hemp shives with expanded perlite, which was used in the HLP1 and HLP2 composites, did not unequivocally influence the value of heat conductivity of the composites. The elevated binder content influences the increase of the apparent density of composite and, thus, the increase of heat conductivity [1]. The amount of hemp aggregate in the compound of composite influences the value of its heat conductivity coefficient; however, it must be noticed that it is not a linear dependence [48]. According to the cited reports, it has been revealed that despite the higher porosity, higher thermal conductivity has been observed in the handmade composites in the wall formworks than in those applied by spraying. It could be also connected with higher heat conductivity of particular compounds—a greater amount of hemp fibers, which are less porous than shives. The work published by [48] presents the value of heat conductivity coefficient for the composite with an apparent density of 377 kg/m^3^ to be equal to 0.089 W/(m·K), and for another composite with an apparent density of 603 kg/m^3^, it is equal to 0.141 W/(m·K). The chart (Figure 5) presents a comparison of the exemplary relations between the lambda coefficient and the apparent density of the hemp-lime composites.

### 3.4. Mass Absorptivity

**Figure 6:**
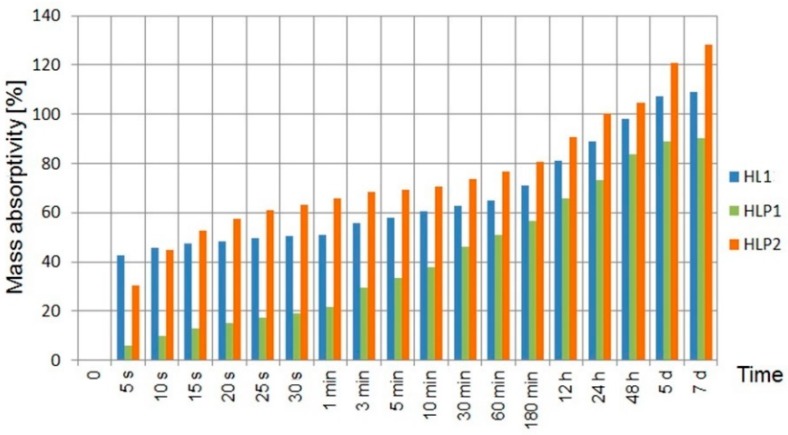
Mass absorptivity of composites.

The graph (Figure 6) presents the results of the tested composites.

The hemp shives themselves are able to absorb water in the amount of twice their own weight in the first 5 s after immersion in water [55]. The largest difference was recorded after the first 5 s since the samples were immersed in water when HL1 reached the absorbability of about 7.3 times higher compared to HLP1 and about 1.4 times higher compared to HLP2. The addition of expanded perlite as a part of the filler proved to be effective. Despite the small difference in the porosity of the samples with and without perlite, the perlite-containing samples obtained a more compact and homogeneous structure, which caused the limitation of water absorption in the first seconds of the study. Similar observations have been described in the literature [56]. Comparing HL1 and HLP1 composites, which contained the same weight ratio of filler to filler equal to 2:1, the perlite-containing composite (HLP1) was characterized by about 20.5% lesser water absorption than the sample containing only the hemp shives as a filler (HL1). The ratio of binder to the filler also had a significant effect on the absorptivity of the composites. The composite with the ratio of binder to the filler 1.5:1 (HLP2) showed greater water absorption than the composite with a ratio of 2:1 (HLP1) by 425% after 5 s and by 42% after 7 days of immersion in water.

The presented test method was designed to simulate extreme conditions—a situation in which part of the wall was flooded for a period of 7 days. Long-term exposure of the hemp-lime composite to water might result in the development of fungi and molds; however, after 7 days of testing, no signs of biological corrosion or other forms of material damage were observed. The research results showed that the greatest increase in the water absorption occurred within the 5 s from the moment of immersion of the samples in water. In practice, this could happen under temporary rainfall.

Water absorptivity strictly depends on the density of the material, which, in turn, is related to the proportion of binder to the shives, as well as their fractions. Stevulova showed [57] that it was possible to limit the absorption of water to about 25%; however, this result was achieved by examining a composite with a bulk density of 1070 kg/m^3^, a fraction of 4–8 mm shives, and a volume composition of shives: binder: water—40:29:31%. Sassoni, in turn, in the studies [58] applied a composite density range from 300 to 1300 kg/m^3^. After 24 h of wetting, the samples with a density of 300 kg/m^3^ showed absorbability of 118.4%, while the composite with the highest density, i.e., 1300 kg/m^3^, showed significantly lower absorbability of 10.1%.

### 3.5. Capillary Uptake Test Results by the TDR Equipment

**Figure 7:**
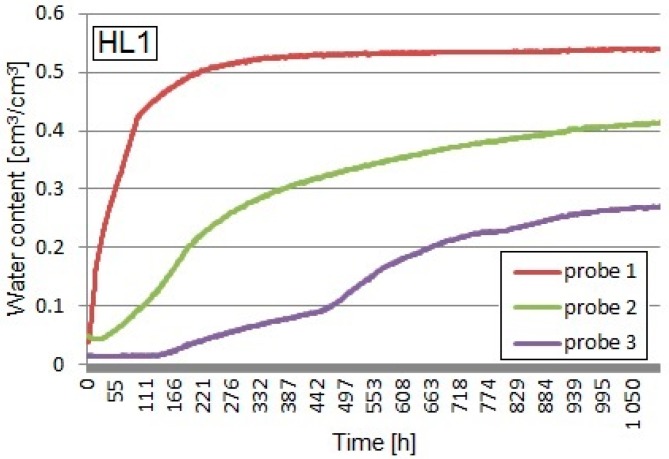
Capillary uptake of the HL1 composite.

**Figure 8:**
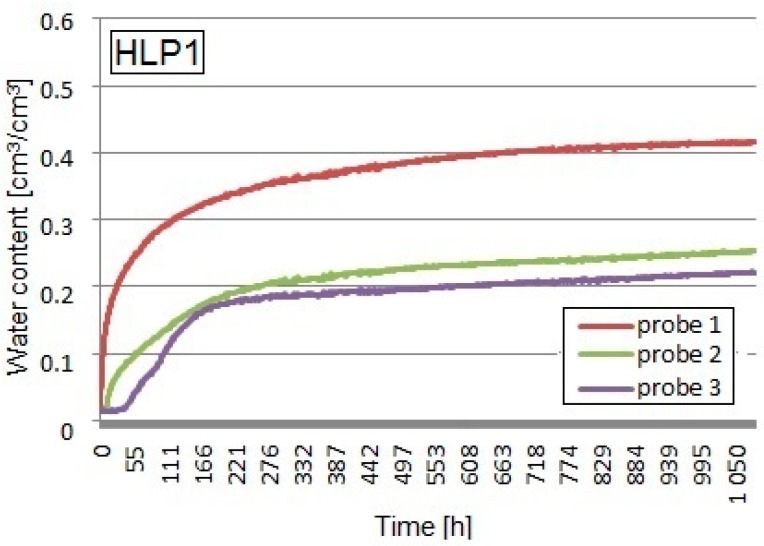
Capillary uptake of the HLP1 composite.

**Figure 9:**
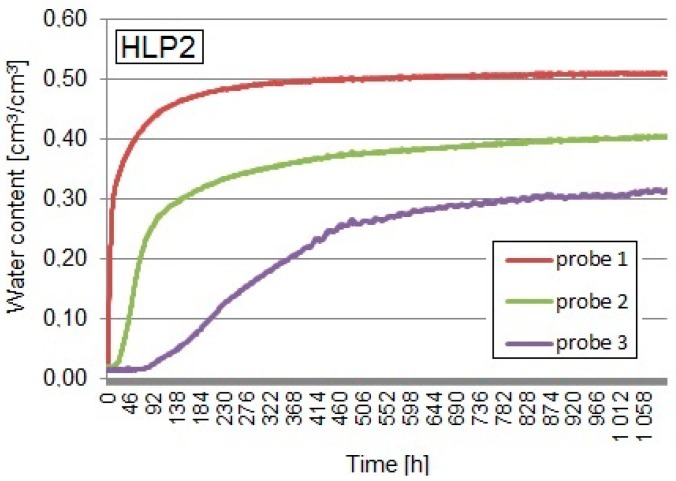
Capillary uptake of the HLP2 composite.

Apparent permittivity determined with the TDR equipment was recalculated into volumetric water content using the Equation (1), taking into consideration the bulk density of the tested composites. The graphs below (Figure 7, Figure 8 and Figure 9) show the volumetric water contents at different levels of the sample at the time intervals obtained from the TDR test on the three tested composites.

The maximum volumetric water content, measured by means of probe No.1 in the HL1 sample, containing the weight ratio of the binder to the filler 2:1 (apparent density of 497.9 kg/m^3^), was equal to 54.2%. In the coverage area of the probe No.2, located 75 mm above the water level, maximum moisture equaled 41.4%, while, in the area of the probe No.3 influence, which was 125 mm above the water level, it amounted to 27.1%. The dynamics of the capillary uptake process in this sample turned out to be the smallest. The moisture of the material in the area of probes No.2 and 3 did not stabilize until the end of the study. The recipe in which the HL1 sample was made using the largest amount of binder contributed to limiting the dynamics of the capillary uptake process (also mass absorption, described earlier), as well as the decrease in the intensity of water uptake at different levels—the moisture in the higher part of the sample (probe No.3) at the end of the test was half the value of the moisture read at the bottom part of the sample (probe No.1). Comparing the results with the HLP1 sample (the same ratio of binder to filler), the presence of expanded perlite decreased material absorptivity but did not slow down the progress in the capillary rise phenomenon.

The maximum volumetric water content, measured by probe No.1 in the HLP1 sample, containing the weight ratio of the binder to the filler 2:1 (apparent density of 503.1 kg/m^3^), was equal to 41.4%. The content of hemp shives reached 60%, and the expanded perlite 40% by weight of the total amount of filler. In the coverage area of the probe No.2, the moisture read was equal to 25.3 vol %, while, in the coverage area of the probe No.3, it was about 21.8 vol %. A dynamic increase in the water content at all measurement levels took place up to the sixth day of the test and was higher compared to the HL1 recipe, but, in the case of the volumetric water content, lower values were achieved.

The maximum volumetric water content, read by the probe No.1 in the sample HLP2, was 52.1%. The weight ratio of the binder to the filler in this recipe was 1.5:1, and the apparent density of the composite was 417.5 kg/m^3^. In the case of the probe No.2, the maximum moisture readouts reached 37.9 vol %, while, in the coverage area of sensor No.3, it was 28.4 vol %. The dynamic moisture growth could be seen in the area of the probes No.1 and 2 influence, in the bottom part of the sample, which was close to the water level. The volumetric water content readouts in the particular areas of the samples measured by the probes No.2 and 3 were stabilized only around the 35th day of the study.

**Figure 10:**
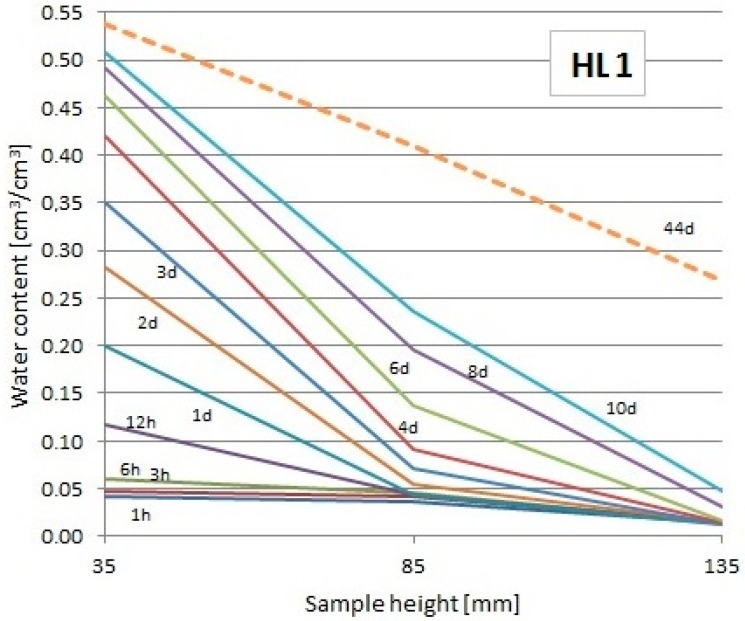
Moisture profiles obtained using the TDR measurement in particular intervals for the HL1 composite.

**Figure 11:**
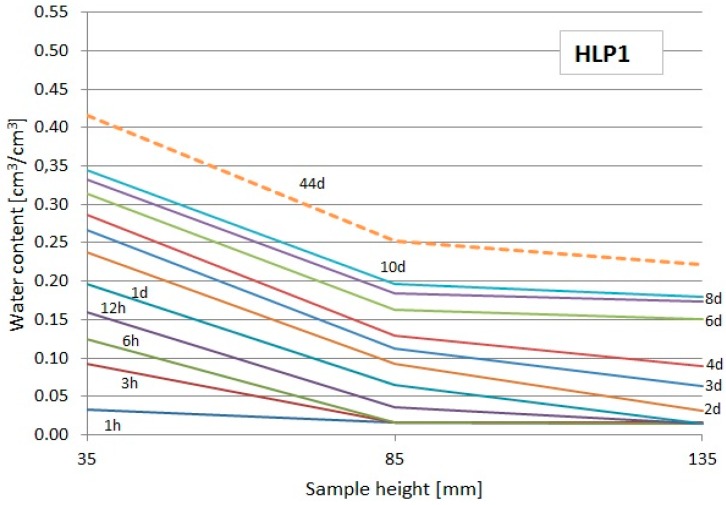
Moisture profiles obtained using the TDR measurement in particular intervals for the HLP1 composite.

**Figure 12:**
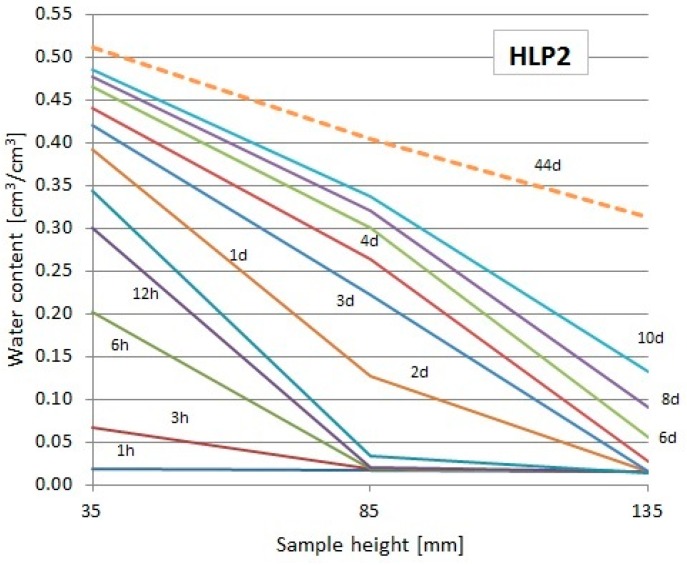
Moisture profiles obtained using the TDR measurement in particular intervals for the HLP2 composite.

The readouts of the used TDR FP/mts probes showed the changes of moisture at particular levels over the water surface, which enabled to draw the moisture profiles at particular periods of time (only first 10 days + final readout). They are presented in Figure 10, Figure 11 and Figure 12. The dashed lines indicate the profiles during the last day of the study.

From the diagrams presented in Figure 10, Figure 11 and Figure 12, it could be noticed that the capillary uptake of moisture in particular composites differed in the particular types of composites. The presented profiles were used to evaluate the water absorption coefficient.

### 3.6. Evaluation of Water Absorption Coefficient Using Standard and Reflectometric Technique

With the moisture profiles obtained with TDR, the mass of absorbed water was calculated according to the following Equation (2). An analogous procedure was used in the studies on autoclaved aerated concrete [59,60]:

(2) $$m=a\cdot b\overset{h}{\underset{0}{\int }}\theta \left(h\right)dh$$

where *m*—mass of absorbed water (g); *a*,*b*—sample cross-section dimensions (width, depth); *h*—sample height; *θ(h)*—moisture characteristics depending on height (moisture profile).

**Figure 13:**
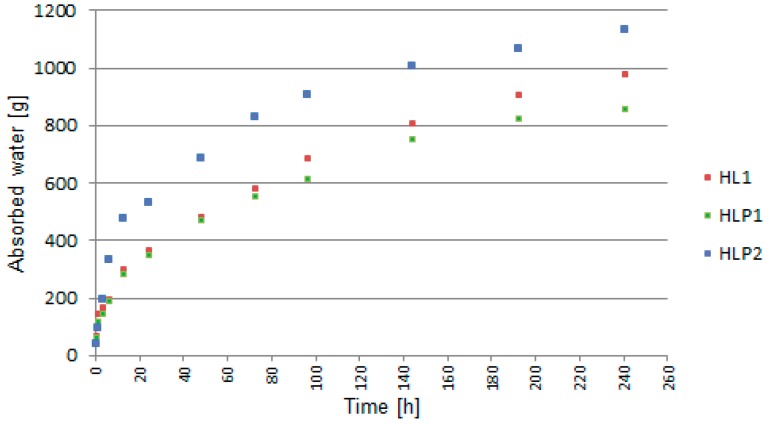
Water absorption by capillary uptake over time using the TDR method.

**Figure 14:**
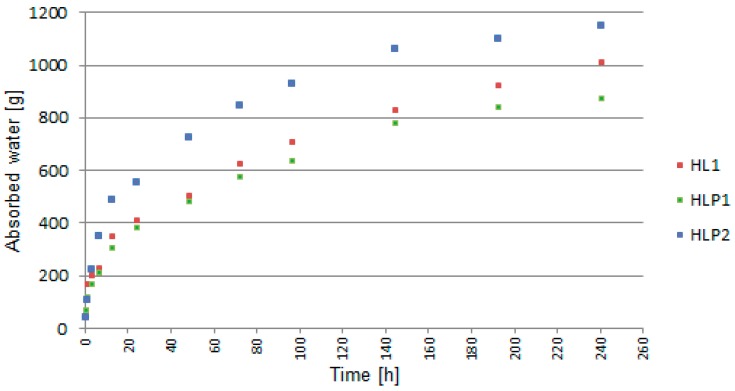
Water absorption by capillary uptake over time, according to the PN-EN 1925 standard method.

The graph (Figure 13 and Figure 14) showed the mass of absorbed water in time intervals examined using the TDR method (Figure 13) and according to the EN 1925 standard (Figure 14).

In both the methods, the tendency of the mass increase of samples was comparable in all tested recipes. The most dynamic water absorption occurred in the first second of contact of the dry sample with water. HLP1 and HL1 had the same binder to filler ratio but differed in the type of fillers, which caused differences in the amount of absorbed water. It could be noticed that the presence of perlite reduced the capillary rising ability of the composite. However, the ratio of binder to filler had a greater effect on the amount of water absorbed. The differences in the rate and magnitude of absorption were noticeable, especially between the group of HLP1 and HLP2 because of the different binder to filler ratio (2:1 and 1.5:1, respectively). A larger amount of binder sealed the pores of hemp shives more effectively. The highest amount of water was absorbed by the HLP2 composite, but showed lower moisture content by volume (Figure 6 and Figure 8) compared to the HL1 composite, which absorbed about 150 g less water. This might be related to the smaller amount of binder contained in HLP2, which resulted in higher porosity. The water was accumulated on the pore walls (as evidenced by the readings from the probes, Figure 8), leaving the pores partially filled with water, thus taking a smaller part sample volume compared to HL1, containing a greater amount of binder.

On the basis of the literature [53], the capillary uptake coefficient was determined after 24 h of testing. The values obtained using the TDR method equaled 2.69, 2.62, and 4.45 kg/m^2^h^1/2^, and, by using the gravimetric method, they were equal to 2.76, 2.68, and 4.53 kg/m^2^h^1/2^ (for HL1, HLP1, and HLP2, respectively). As visible in the diagram, the HL1 and HLP1 composites were characterized by the similar values of the evaluated parameter. In the case of the HLP2 composite, the capillary uptake coefficient value was significantly higher and amounted to over 4 kg/m^2^h^1/2^. This was mainly caused by the lower share of the binder compared to the other recipes, and the air gaps between the shives were larger, and thus the absorptivity of the material was higher. Moreover, it should be noticed that the coefficient values determined using the TDR technology are underestimated for about 2% on average.

In the literature, there are reports on the capillary uptake of water examination on hemp-lime composites. They are mainly based on the EN 1925 standard, rather than the reflectometric techniques. Such an experiment has been described in [53], where the ability of capillary uptake of water by the lime-hemp composite with different recipes that differ in the binder and the weight ratio between binder and aggregate 2:1 has been described. The capillary uptake coefficient has been in the range between 2.65 and 3.37 kg/m^2^h^1/2^ within the first 24 h since dipping. The authors stated that the kind of a binder did not significantly influence the readouts; therefore, only one kind of a binder was used in the next examinations. In [61], it has been stated that the addition of methylocellulose to a binder influences the limitation of the capillary uptake phenomenon because it may create small pores in a binder that cut the path for the water to be raised.

The research of the capillary rise on the lime-hemp composite has also been presented in the research [62]. The composites that differed in the binder configuration (hydrated lime, hydraulic lime, Portland concrete) and weight ratio between shives and the binder (between 0.22 and 0.33) have been used for the investigation. In their research, the authors revealed no significant differences in the behavior of the material produced using all of the applied recipes. The average coefficient of capillary uptake is reported as 9 kg/m^2^h^1/2^ (0.15 kg/m^2^s^1/2^).

**Figure 15:**
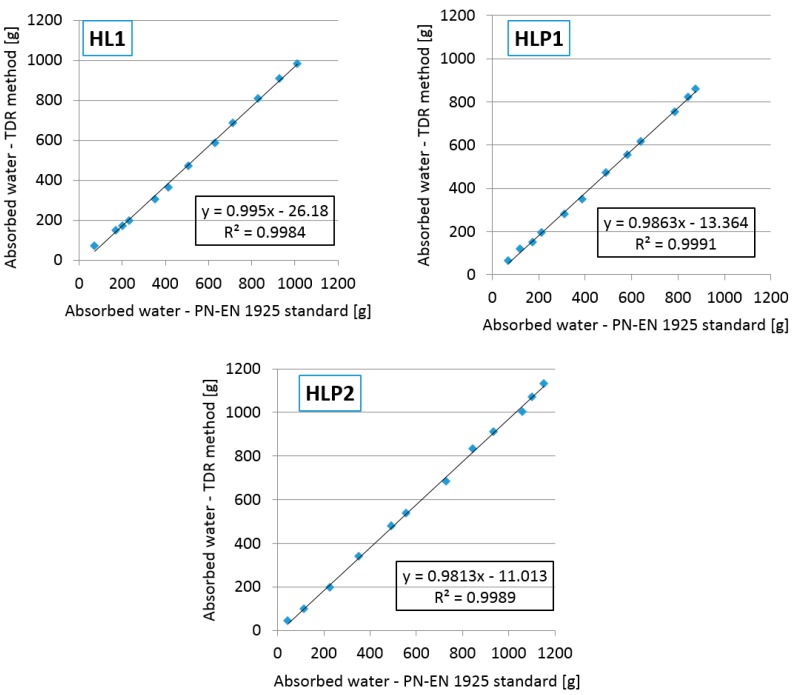
Correlations between the amount of absorbed water evaluated using the gravimetric method and the reflectometric one.

In order to determine the similarity of the results of gravimetric and reflectometric tests, the graphs presented in Figure 15 were prepared to show the correlation.

The differences in the results of the test performed with the two methods were not significant. The results obtained with the gravimetric method were 2.3–14.2% higher for the HL1 composite, 2.1–13.4% for HLP1, and 1.3–12.6% for HLP2, in comparison with the results obtained by means of the TDR method in the adopted time intervals. Nevertheless, the correlation between the processes measured using both techniques was high. The dependences between the moisture readouts using both techniques of detection were linear, which was proven by the formulas evaluated in Figure 15 and coefficients of determination R^2^ reaching over 0.99 in each case. Moreover, the values of slopes of linear formulas equaled between 0.981 and 0.995 and were close to 1. On the other hand, the y-intercept values of the linear correlation models were negative and between −11.01 and −26.18, which meant that the reflectometric readouts were lower in the moisture value compared to the gravimetric evaluation.

## 4. Conclusions

The research presented the possibilities of using hemp shives and expanded perlite as an aggregate of an eco-friendly composite based on a lime binder for use as wall filling. The basic properties of composites and their ability of capillary uptake of water were investigated.

A thorough analysis of the capillary uptake results enabled to formulate the following conclusions:

- The maximum volumetric water content in the tested composites was from 41.4 to 54.2 cm^3^/cm^3^ (based on the TDR method). The highest volumetric water content of the sample was demonstrated by the HL1 composite, while the smallest by the HLP1 composite.
- The maximum volumetric water content measured using the TDR equipment nearly reached the value of volume absorptivity of all samples determined in pre-tests. On average, the reflectometric readouts were underestimated for 2.5–3.5% (HLP2 and HL1 samples) and for 9.4% (HLP1 sample).
- Mass of the absorbed water determined with the gravimetric method was higher by 1.3–3.0% in comparison with the results obtained using the TDR method. The HL1 sample showed the largest differences, whereas HLP2 showed the smallest.
- In general, the Time Domain Reflectometry readouts of the moisture properties of the examined composites were underestimated for about 2% compared to the gravimetric evaluation. This could be the consequence of the applied universal calibration model but also measuring uncertainty.
- The ratio of binder to filler had a greater effect on limiting the water absorption due to the capillary uptake than the kind of filler.
- Comparing composites with the same ratio of binder to filler (HL1 and HLP1), less water absorption due to the capillary uptake was exhibited by the composite containing expanded perlite (about 14.5% lower).
- The HL1 and HLP1 composites were characterized by similar values of the water absorption coefficient values (2.62–2.76 kg/m^2^h^1/2^ based on both methods of testing). In the case of the HLP2 composite, the coefficient value was significantly higher and amounted to over 4 kg/m^2^h^1/2^.
- The mass absorptivity of composites was 90.1–127.8%. The most dynamic water absorption occurred in the first seconds after the immersion of samples in water. The absorptivity of the HL1 sample after 5 s was 42.7%. The presence of expanded perlite resulted in a reduction of the initial water absorption.

On the basis of the test results of other measured properties, the following conclusions were drawn:

- Composites had an apparent density of 417.5–503.1 kg/m^3^. Partial replacement of hemp shives with perlite reduced the density by about 1%. Lowering the proportion of binder to filler from 2:1 to 1.5:1 reduced the density by 20.5%.
- Composites were characterized by low values of flexural (0.16–0.23 MPa) and compressive strength (0.55–0.78 MPa). The highest parameters were shown by the HLP1 composite, while the smallest by the HLP2 composite. Partial replacement of the shives with perlite resulted in an increase in the strength parameters.
- Hemp-lime composites were characterized by the thermal conductivity in the range of 0.087–0.111 W/(m·K). Partial replacement of hemp shives with perlite affected the difference in the thermal conductivity coefficient of the HL1 sample to a limited extent; the sample had an average lambda value lower by 2.8% than HLP1.

## Figures and Tables

**Table 1:** Mixture amounts (by weight).

Material	Binder	Hemp Shives	Expanded Perlite	Water
symbol/unit	(kg/m^3^)	(kg/m^3^)	(kg/m^3^)	(kg/m^3^)
HL1	284.44	142.22	-	412.44
HLP1	334.44	101.33	65.87	484.88
HLP2	250.80	101.33	65.87	413.82

**Table 2:** Mixture proportions (by weight).

Material/Symbol	Binder	Hemp Shives	Expanded Perlite	Binder: Water Ratio
HL1	2	1	-	1.45
HLP1	2	0.6	0.4	1.45
HLP2	1.5	0.6	0.4	1.65

**Table 3:** Mechanical and physical properties of composites (average value ± SD).

Recipe Symbol	Flexural Strength	Compressive Strength	Apparent Density	Total Porosity	Open Porosity	Mass Absorptivity	Volume Absorptivity	Thermal Conductivity Coefficient
	(MPa)	(MPa)	(kg/m^3^)	(%)	(%)	(%)	(%)	(W/(m·K))
HL1	0.18	0.66	497.9	74.4	57.8	112.6	56.1	0.108
	±0.018	±0.019	±5.25	±0.451	±0.251	±4.52	±2.35	±0.004
HLP1	0.23	0.78	503.1	75.6	49.9	90.1	45.3	0.111
	±0.026	±0.025	±6.35	±0.491	±0.203	±3.93	±2.02	±0.005
HLP2	0.16	0.55	417.5	79.3	54.3	127.8	53.4	0.087
	±0.013	±0.014	±5.80	±0.547	±0.242	±5.48	±2.37	±0.002
